# The Early Season Community of Flower-Visiting Arthropods in a High-Altitude Alpine Environment

**DOI:** 10.3390/insects13040393

**Published:** 2022-04-16

**Authors:** Marco Bonelli, Elena Eustacchio, Daniele Avesani, Verner Michelsen, Mattia Falaschi, Marco Caccianiga, Mauro Gobbi, Morena Casartelli

**Affiliations:** 1 Department of Biosciences, University of Milan, 20133 Milan, Italy; elena.eustacchio@unimi.it (E.E.); marco.caccianiga@unimi.it (M.C.); morena.casartelli@unimi.it (M.C.); 2 Research and Museum Collections Office, Climate and Ecology Unit, MUSE—Science Museum, 38122 Trento, Italy; mauro.gobbi@muse.it; 3 Zoology Section, Civic Museum of Natural History of Verona, 37129 Verona, Italy; danieleavesani@yahoo.it; 4 Natural History Museum of Denmark, University of Copenhagen, DK-2100 Copenhagen, Denmark; vmichelsen@snm.ku.dk; 5 Department of Environmental Science and Policy, University of Milan, 20133 Milan, Italy; mattia.falaschi@unimi.it; 6 Interuniversity Center for Studies on Bioinspired Agro-Environmental Technology (BAT Center), University of Naples Federico II, 80138 Naples, Italy

**Keywords:** *Androsace brevis*, Alps, biotic interactions, Diptera Anthomyiidae, Hymenoptera, insect pollinators, mountain ecosystems, parasitoid wasps, temperature, wind speed

## Abstract

**Simple Summary:**

The knowledge about the flower-visiting arthropods in high-altitude environments is limited, in particular about those occurring on early flowering plants. We characterised the flower visitor community of an early flowering high-altitude Alpine species: *Androsace brevis*, a vulnerable endemic plant belonging to the Primulaceae family, which grows in the Alps above 2000 m asl and flowers for a very short period immediately after snowmelt. In addition, we tested the effect of temperature, wind speed, and other variables on flower-visiting arthropod activity. We identified dipterans (in particular, anthomyiid flies) and hymenopterans (in particular, ants and parasitoid wasps) as the main flower visitors. Moreover, we assessed that temperature and time (hour of the day) affect the flower visitors’ activity. Our study contributes to defining the composition of high-altitude Alpine flower-visiting arthropod communities and sets the stage for future evaluation of climate change effects on flower-visiting arthropods in high-altitude environments in the early season.

**Abstract:**

In mountain ecosystems, climate change can cause spatiotemporal shifts, impacting the composition of communities and altering fundamental biotic interactions, such as those involving flower-visiting arthropods. On of the main problems in assessing the effects of climate change on arthropods in these environments is the lack of baseline data. In particular, the arthropod communities on early flowering high-altitude plants are poorly investigated, although the early season is a critical moment for possible mismatches. In this study, we characterised the flower-visiting arthropod community on the early flowering high-altitude Alpine plant, *Androsace brevis* (Primulaceae). In addition, we tested the effect of abiotic factors (temperature and wind speed) and other variables (time, i.e., hour of the day, and number of flowers per plant) on the occurrence, abundance, and diversity of this community. *A. brevis* is a vulnerable endemic species growing in the Central Alps above 2000 m asl and flowering for a very short period immediately after snowmelt, thus representing a possible focal plant for arthropods in this particular moment of the season. Diptera and Hymenoptera were the main flower visitors, and three major features of the community emerged: an evident predominance of anthomyiid flies among Diptera, a rare presence of bees, and a relevant share of parasitoid wasps. Temperature and time (hour of the day), but not wind speed and number of flowers per plant, affected the flower visitors’ activity. Our study contributes to (1) defining the composition of high-altitude Alpine flower-visiting arthropod communities in the early season, (2) establishing how these communities are affected by environmental variables, and (3) setting the stage for future evaluation of climate change effects on flower-visiting arthropods in high-altitude environments in the early season.

## 1. Introduction

Climate change constitutes a key threat to biodiversity [1,2,3,4,5] and can strongly impact mountain ecosystems [6,7], affecting single species but also causing altitudinal and phenological shifts that can alter both the composition of communities and biotic interactions [8,9,10]. In particular, some recent studies focussed on potential mismatches between flowering plants and flower-visiting arthropods in mountain environments [11,12,13,14]. Moreover, climate-change-driven mismatches can impact the interactions among arthropods, such as those between host and parasitoid or prey and predator [15], which frequently occur on flowers [16,17,18,19,20,21], but to the best of our knowledge, no studies investigated potential mismatches among flower-visiting arthropods in mountain ecosystems. One of the main problems in assessing the effects of climate change on biodiversity is the lack of robust data about species occurrence and diversity [22]. Furthermore, the available information comes mostly from human-altered ecosystems and thus is not useful for drawing conclusions about natural ones [22,23]. Therefore, it is essential to collect precise, current data to build a solid platform of knowledge that can help develop further comparative studies and proper conservation plans in the near future.

The Alps are the highest and longest mountain range that lies entirely in Europe, and they host peculiar biodiversity, comprising several endemic plants and arthropods and threatened species [24,25,26,27,28,29]. While a significant body of literature deals with plant diversity, species occurrence, and phenology in the Alps (e.g., [30,31,32,33,34,35]), not many studies have addressed the topic of flower-visiting arthropod communities and abiotic factors impacting their activity in this environment [36,37,38,39,40,41,42,43,44,45]. The paucity of information on arthropods is a global issue in studying mountain ecosystems [46,47], although arthropods are the most abundant and diverse group of organisms [48,49] and play crucial roles in terrestrial ecosystem functioning, especially through interactions with plants [50,51,52,53,54,55,56]. In addition, many faunistic surveys about flower-visiting arthropods are based on data that were collected without applying standardised and repeatable methods, making it difficult or even impossible to use them for future analyses and evaluations [22,36]. Finally, although environmental variables such as temperature and wind speed can impact flower visitors’ activity [57,58], only a few studies reported the micrometeorological conditions occurring during the survey, thus often preventing any evaluation of the context in which the data were collected. This lack of information can be particularly problematic in mountain environments where extreme weather changes and unstable conditions can be observed even in the same period of the season. Therefore, there is a need to collect reliable data on arthropods in mountain ecosystems, which will be integral for future studies assessing their response to climate change [59,60], as well as to increase our knowledge of high-altitude flower-visiting arthropods

These considerations prompted us to perform a first assessment of the flower-visiting arthropod community in a high-altitude Alpine environment, using the narrow-endemic plant *Androsace brevis* (Hegetschw.) Cesati (Primulaceae) as a model species. Climate warming could represent a serious threat for this species, as it is almost impossible to shift its range upward since the plant already lives on mountain ridges and tops. Moreover, *A. brevis* shows low competitive ability, and the upward shift of more competitive species from a lower altitude could represent a serious threat [61]. The flowering period of *A. brevis* is very short and occurs in the early season, when snow cover is still present, except for ridges and outcrops, and very few other floral resources are available for arthropods [45]. In this context, *A. brevis* flowers can thus represent a possible focal species for arthropods. Moreover, the early season is particularly interesting because it is a critical moment for possible mismatches between plants and flower-visiting arthropods [62,63] and phenological shifts in early spring can occur more rapidly than later in the season [64].

Here, we present data collected over four years on *A. brevis* flower-visiting arthropods, together with the micrometeorological conditions that occurred during sampling.

The goal of our study was to shed light on the composition and response to environmental variables of flower-visiting arthropods of an early flowering high-altitude Alpine plant. We performed an omni-comprehensive assessment considering all flower-visiting-arthropods, not only those having a possible role as pollinators and/or interacting with flowers for feeding activity but also those present for other purposes (e.g., for mating, sheltering, basking, or finding a prey) or by chance, highlighting possible biotic interactions in which these arthropods are involved. Our data can be useful to develop a platform of knowledge suitable for future evaluation of the effects of climate change on early season Alpine flower-visiting communities.

## 2. Materials and Methods

### 2.1. Study Species

**Figure 1:**
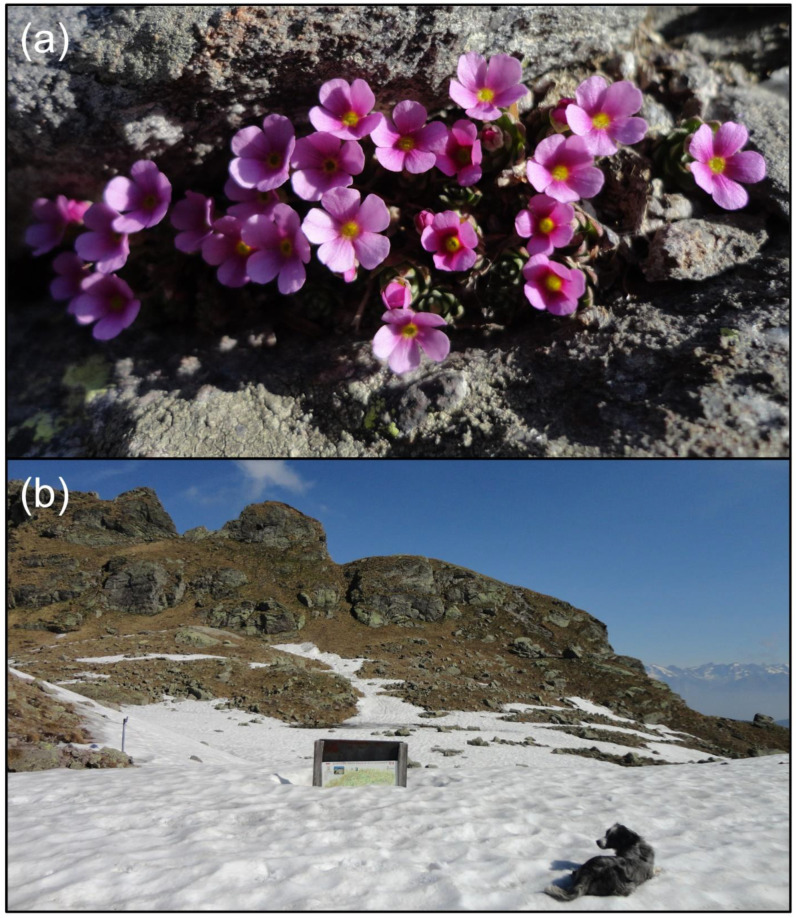
*Androsace brevis* (**a**) and snow cover during its flowering at the Mountain Hut ‘Cesare Benigni’ in the Orobic Alps (Bergamo, Lombardy) on 31 May 2017 (**b**).

*Androsace brevis* (Figure 1a) is a narrow-endemic cushion plant that grows above 2000 m asl on rocky ridges and outcrops in a limited area in the southern Alps of northern Italy (Lombardy) and neighbouring Switzerland, in few and scattered populations of limited size. Its conservation status in Italy is vulnerable (VU) according to IUCN criteria [61]. The flowering period is very short, typically lasting about 2 weeks between the end of May and the beginning of June. It occurs where the snow has just melted, while in the immediate vicinity, it is still present in patches (Figure 1b). Each plant carries from a few to about 200 solitary flowers (ca. 4 mm long) with a pink corolla (ca. 8 mm in diameter) and a yellow mouth (ca. 0.9 mm in diameter); flowers are held by 0.5–2 cm erect pedicels [45,61].

### 2.2. Study Sites

**Figure 2:**
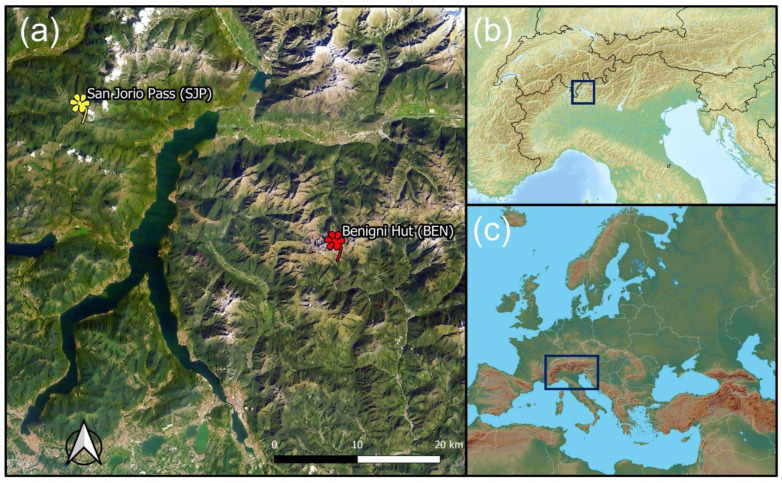
Study sites (**a**) and their location in the Alps (**b**) and in Europe (**c**).

The present study was conducted in the Alpine biogeographical region [65] in the southern Alps of northern Italy. Two sites were selected across the *A. brevis* distribution range: San Jorio Pass—Cima di Cugn (SJP) in the Lepontine Alps (Como, Lombardy) and Mountain Hut ‘Cesare Benigni’ (BEN) in the Orobic Alps (Bergamo, Lombardy) (Figure 2). 

At the first site (SJP), the fieldwork was conducted on an *A. brevis* population located along the ridgeline north of the San Jorio Pass and southwest of Cugn Peak (UTM WGS84—32T E 512338 N 5112905, 2193 m asl), close to the Mountain Huts ‘Rifugio San Jorio’ and ‘Capanna delle Aquile’. The site is characterised by a continental climate without a dry season [66]. In particular, the mean annual temperature is 6.2 °C with a minimum of −1.9 °C in February and a maximum of 16.0 °C in August. The average annual rainfall amounts to about 1800 mm, mostly concentrated in the equinoctial months (extrapolated from data of the climatic stations: ‘Cavargna’, 1100 m asl, 8.9 km from the study site, observation period 2012–2020; ‘Porlezza’, 280 m asl, 13.4 km from the study site, observation period 2014–2020; ‘Garzeno’, 688 m asl, 8.1 km from the study site, observation period 2013–2020). 

At the second site (BEN), located within the Orobie Bergamasche Regional Park, the fieldwork was conducted near the Mountain Hut ‘Cesare Benigni’ (UTM WGS84—32T E 543496 N 5096577, 2222 m asl). The site is characterised by a temperate climate with a humid summer [66]. In particular, the mean annual temperature is 3.3 °C, with a minimum of −4.2 °C in February and a maximum of 11.4 °C in July; the average annual rainfall amounts to about 1800 mm, distributed throughout the year (extrapolated from data of the climatic stations: ‘Gerola Alta Pescegallo’, 1875 m asl, 1.5 km from the study site, observation period 2012–2020; ‘Mezzoldo Passo San Marco’, 1824 m asl, 5.5 km from the study site, observation period 2012–2020; ‘Valtorta’, 982 m asl, 5 km from the study site, observation period 2012–2020). 

Snow cover usually lasts from October/November to May/June at both sites, although the snow generally melts in SJP about 1 week sooner than in BEN. To evaluate the accessibility of both sites and estimate the full flowering period of *A. brevis*, snow cover was monitored daily by the Mountain Hut ‘Cesare Benigni’ webcam (available at https://orobiemeteo.com/ (accessed on 1 April 2022)).

The fieldwork was conducted for 2 years at each site (SJP: 2016 and 2019, BEN: 2017 and 2018) during the *A. brevis* flowering period. During the fieldwork period, a maximum of 13 entomophilous plants other than *A. brevis* were flowering at the study sites.

### 2.3. Sampling of Flower-Visiting Arthropods

For each year and site, two focal plants of *A. brevis*, more than 5 m apart and bringing more than 20 flowers at anthesis each, were randomly selected. On these plants, flower-visiting arthropods were sampled according to the timed-observation method [67], as described in Bonelli et al. [45]. Briefly, two simultaneous sampling sessions, lasting 1 h, were conducted, with two different pairs of operators observing the two focal plants during the same time windows (Table 1). Time windows were interspersed with a break lasting from 30 to 60 min, to reduce the disturbance to flower-visiting arthropods and to ensure the independence of data points obtained from the same plant on the same day. The operators collected in 70% ethanol all flower-visiting arthropods (i.e., individuals touching at least one flower of the plant). The operators were crouched at opposite sides of the plant, 50 cm apart, thus having a clear view of the flowers while minimising disturbances for the flower visitors. Each year, we conducted two days of sampling, with four pairs of sampling sessions per day, for a total of 16 h of timed observations per year, except for 2019 at SJP, when the extreme meteorological conditions (i.e., hailstorm) hampered the fieldwork. In total, we performed 54 sampling sessions, corresponding to 54 h of flower-visiting arthropod sampling by timed observations (Table 1). Achieving these total cumulative hours of timed observations should be considered relevant since the amount of time available for sampling is constrained by multiple factors: the very short flowering period, occurring when snow cover is still partially present and microclimatic instability is possible, and the remoteness of the sites.

During the timed observations, we recorded the air temperature at ground level near the selected plants and the wind speed at 50 cm from the ground using data loggers (Tinytag Plus 2) and thermo-anemometers (LaCrosse Technology EA-3010U), respectively. Moreover, to increase the volume of data on flower-visiting arthropods, free observation sampling was conducted on the same days and sites of timed observation sampling but at least 10 m away from focal plants that were considered for the timed observations. In this case, operators walked freely in the study site, observing many different *A. brevis* plants, and collecting in 70% ethanol all the arthropods they saw on flowers. These data were not considered in any statistical analysis and were only used to provide a broader description of the flower visitors’ diversity.

### 2.4. Identification of Flower-Visiting Arthropods

The ethanol-preserved specimens were shipped for morphological identification to expert taxonomists (Table S1), who were allowed to keep them for private or institutional collections. For insects whose morphological identification was particularly challenging or not possible (Table S2), molecular identification was performed through COI barcoding. The arthropods were rinsed in phosphate-buffered saline (137 mM NaCl, 2.7 mM KCl, 8.1 mM Na_2_HPO_4_, 1.8 mM KH_2_PO_4_, pH 7.4), and the genomic DNA was extracted using the CTAB protocol [68], as adapted to insect samples by Bonelli et al. [69]; then, a fragment of the mitochondrial COI gene was amplified. The primer sets used and their references, PCR conditions, and the size of the amplicons are reported in Table S2. A commercial service provider (Eurofins Genomics, Vimodrone, Italy) purified and sequenced the PCR products. All sequences were uploaded to GenBank (Accession numbers are provided in Table S2). The resulting sequences were queried against the GenBank database (NCBI) using the basic local alignment search tool (BLAST) and against the Barcode of Life Data System (BOLD) using the identification engine with a species-level option. Species identity was assigned when the similarity statistic (the number of nucleotide identities between the query and reference) was > 99%.

### 2.5. Statistical Analyses

Differences in the mean of the micrometeorological data among the four years of sampling were assessed with ANOVA for wind speed and Kruskal–Wallis test for temperature (as for temperature, ANOVA assumptions were not fulfilled: residuals not normally distributed, Shapiro–Wilk test *p* < 0.05). The influence of micrometeorological conditions (i.e., temperature and wind speed, as means of the values recorded during the sampling session) on flower visitors’ presence (i.e., presence/absence of sampled specimens during the sampling session), diversity (i.e., the total number of different families sampled during the sampling session) and abundance (i.e., the total number of sampled specimens during the sampling session) was tested using generalised linear models (GLMs) with binomial (for presence) and Poisson (for diversity and abundance) distribution. The time (both linear and quadratic terms) and the number of flowers per plant were included as covariates in the model, while the site was included as a fixed factor. Time was indicated as the hour of the day, expressed in minutes lasting from midnight to the middle of the considered sampling session (e.g., for a sampling session performed in the time window 11.30–12.30, time was indicated in the model as 720 min, that is time in minutes from midnight to the middle of the time window—i.e., 12.00). Each data point corresponds to a single sampling session (Table 1); therefore, all dependent and independent variables were calculated for each sampling session. All continuous independent variables were standardised before analyses with a mean of 0 and a standard deviation of 1. The same analyses were performed considering both all the flower visitors sampled during the timed observations and only the flying visitors (defined as the arthropods with functional wings); the analyses on flying arthropods were performed to test whether the variables (e.g., wind) affect their activity specifically. The ‘*glm’* and ‘*drop1*’ functions from the *stats* package [70] were used to perform the analysis and obtain the *p*-value for each independent variable considered, and the *visreg* package [71] was used to generate plots. Before running the models, we calculated Pearson’s correlation coefficient between all pairwise combinations of independent variables using the ‘*cor*’ function from the *stats* package [70]. Correlation coefficients were always <|0.7|; hence, all the independent variables were kept in the models. Model performances were evaluated through a likelihood ratio test and by calculating the pseudo R^2^, performed with the *‘anova’* function from the *stats* package [70] and with the ‘*r2*’ function from the *performance* package, respectively [72]. All statistical analyses were performed in an R environment (R version 4.1.0).

## 3. Results

All sampled specimens were identified to order level, 99% to family, 84% to genus, and 71% to species level (Tables S3 and S4).

During the timed observations, 140 arthropods were sampled, with 2.6 ± 0.3 captures per hour (mean ± SEM), for a total of 9 orders and 33 families (Table 2 and Table S3), with 26 identified species (Table S3).

**Figure 3:**
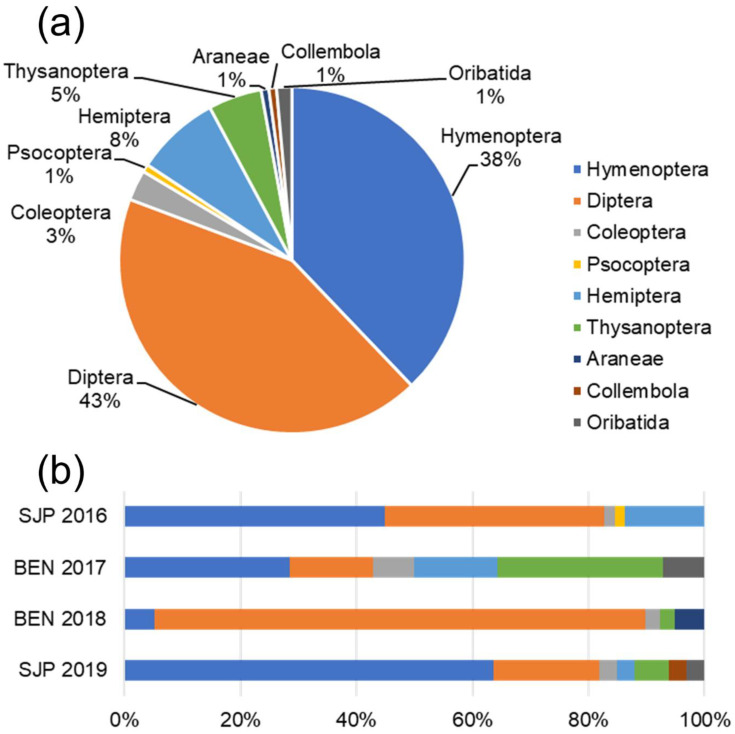
Arthropods sampled by timed observations: overall (**a**) and yearly composition at the two sites (**b**) of the flower-visiting arthropod community to order level.

The two most abundant taxa sampled during timed observations were Diptera (43%) and Hymenoptera (38%), followed by Hemiptera (8%), Thysanoptera (5%), and Coleoptera (3%); other arthropod orders were each represented by about 1% of the total individuals sampled (Figure 3a, Table S3). In particular, Diptera and Hymenoptera represented more than half of the flower-visiting arthropods for all years and sites (Figure 3b).

Among Diptera, 45% of the species belonged to the family Anthomyiidae, followed by Sphaeroceridae (13%), Sciaridae (12%), Chironomidae (10%), and Chloropidae (5%), while Agromyzidae, Anthomyzidae, Cecidomyiidae, Drosophilidae, Lonchopteridae, Phoridae, and Sepsidae were each represented by less than 5% of the sampled Diptera (Table S3). Regarding Hymenoptera, the sampled specimens belonged to the families Formicidae (45%), Braconidae (26%), and Eulophidae (6%); the sample rates for Ceraphronidae, Encyrtidae, Figitidae, Ichneumonidae, Megaspilidae, Mymaridae, Pteromalidae, Scelionidae, and Torymidae were all less than 5%. All the identified Hemiptera belonged to Aphididae, and all Thysanoptera to Thripidae. Coleoptera were represented by Chrysomelidae, Coccinellidae, Meloidae, and Staphylinidae (25% each, one specimen each family). For Collembola, Psocoptera, and Araneae, a single specimen was sampled for each order, belonging to the families of Entomobryidae, Ectopsocidae, and Linyphiidae, respectively.

In addition to the arthropods sampled during timed observation, more than 100 flower visitors were sampled during free observations (24 in 2016 at SJP, 25 in 2017 at BEN, 61 in 2018 at BEN, and four in 2019 at SJP) (Table S4). They belonged to eight orders (Araneae, Collembola, Coleoptera, Diptera, Hemiptera, Hymenoptera, Lepidoptera, and Thysanoptera), and 30 families, with 28 identified species (Table S4). By applying this method, we could identify 1 order (Lepidoptera) and 13 families of flower visitors not detected during timed observations: Diptera Ephydridae, Muscidae, Syrphidae, and Scathophagidae; Hymenoptera Andrenidae, Apidae, Halictidae, and Tenthredinidae; Coleoptera Cantharidae and Curculionidae; Lepidoptera Nymphalidae; Hemiptera Cicadellidae; Araneae Theridiidae.

Overall, considering both timed and free observations, 254 flower-visiting arthropods belonging to 10 orders and 47 families were sampled.

The daily patterns of temperature (Figure S1a,c,e,g) and wind speed (Figure S1b,d,f,h) varied between samplings; however, no significant difference was observed in mean temperature (Kruskal–Wallis test, *p* = 0.1295; Figure S1i) and mean wind speed (ANOVA, *p* = 0.0561; Figure S1j) among the four years.

The models relating micrometeorological conditions to flower visitors’ presence, abundance, and diversity showed better performance than null models and good R-squared values (Table S5).

**Figure 4:**
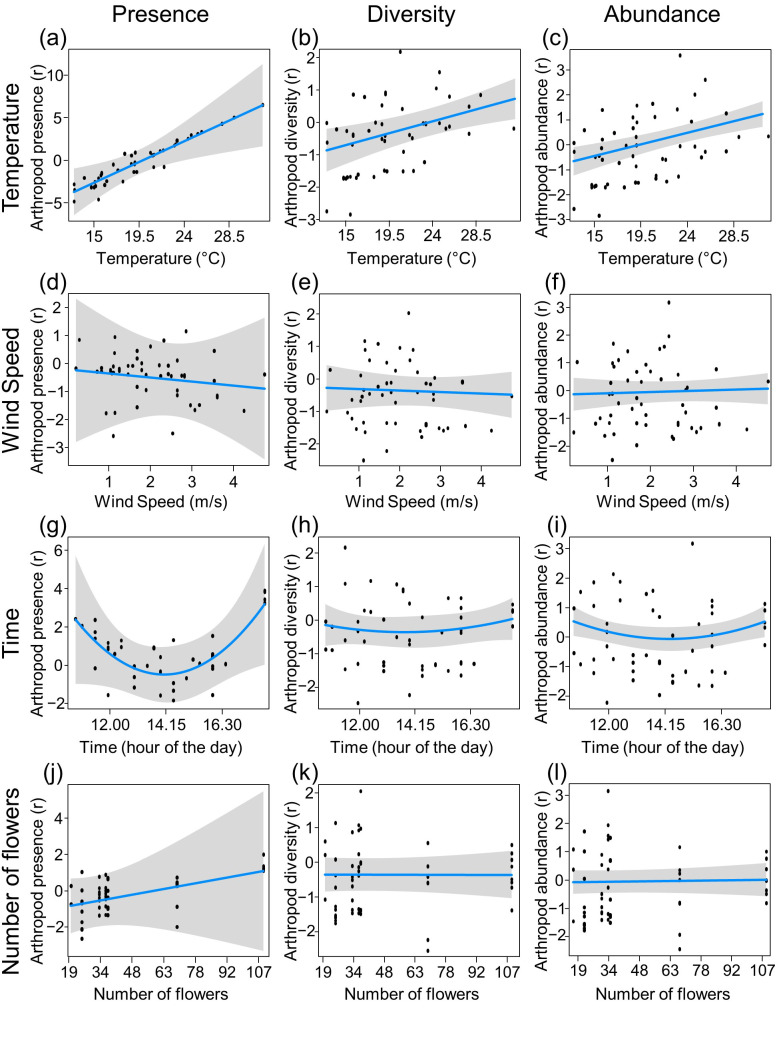
Partial residual plots showing the influence of temperature (**a**–**c**), wind speed (**d**–**f**), time (quadratic) (**g**–**i**), and number of flowers (**j**–**l**) on the presence (**a**,**d**,**g**,**j**), diversity (**b**,**e**,**h**,**k**) and abundance (**c**,**f**,**i**,**l**) of flower-visiting arthropods. Each black dot represents a sampling session. Blue lines indicate the mean value obtained using GLMs, while shaded areas represent 95% confidence bands; r = residuals.

The temperature had a significant, positive effect on the presence (χ^2^_1_ = 17.88, *p* < 0.001), diversity (χ^2^_1_ = 10.38, *p* < 0.01) and abundance (χ^2^_1_ = 20.49, *p* < 0.001) of flower visitors (Figure 4a–c), while the wind speed did not significantly affect any of the dependent variables considered (presence: χ^2^_1_ = 0.08, *p* = 0.775; diversity: χ^2^_1_ = 0.14, *p* = 0.705; abundance: χ^2^_1_ = 0.19, *p* = 0.666) (Figure 4d–f). Regarding covariates, the quadratic time showed a significant effect on the presence (χ^2^_1_ = 4.81, *p* < 0.05) and abundance (χ^2^_1_ = 5.43, *p* < 0.05) of flower visitors but not on their diversity (χ^2^_1_ = 0.96, *p* < 0.326) (Figure 4g–i), whereas the number of flowers did not have any significant effect on the presence (χ^2^_1_ = 0.65, *p* = 0.421), diversity (χ^2^_1_ = 0.00, *p* = 0.999), and abundance (χ^2^_1_ = 0.09, *p* = 0.764) of flower visitors (Figure 4j–l). The site significantly affected their presence (χ^2^_1_ = 3.88, *p* < 0.05), diversity (χ^2^_1_ = 28.57, *p* < 0.001), and abundance (χ^2^_1_ = 30.32, *p* < 0.001). Also considering only the flying flower visitors, temperature had a significant effect on the dependent variables considered (presence, diversity and abundance), whereas wind did not have any significant effect (Table S6).

## 4. Discussion

This research provides new insights into the flower-visiting arthropods of an early flowering high-altitude Alpine plant (*Androsace brevis*), taking into consideration the effect of abiotic factors (temperature and wind speed) and other variables (time and number of flowers) on their occurrence, abundance, and diversity. 

The most abundant order of flower visitors (i.e., all the arthropods occurring on *A. brevis* flowers) was Diptera, nearly half of which were flies belonging to Anthomyiidae. This family was represented almost entirely by *Paregle coerulescens* (Strobl, 1893) and *Delia platura* (Meigen, 1826). The first insect is a common high-altitude species in central and southern Europe; the second one lives in temperate areas throughout the world and is known to feed on flowers [73]. Therefore, the flower visits by Anthomyiidae can be due to trophic activity, as actually observed on *A. brevis*, and in previous reports on other plant species [74,75]. Moreover, anthomyiid flies may visit flowers for thermoregulation, spendingtime on sun-exposed flowers, as reported in the literature for other anthophilous Diptera [76,77]. This thermoregulatory behaviour can be particularly relevant in cold environments [78], and climate change might negatively impact it, leading to fewer visits to flowers in the case of higher temperatures, thus reducing the pollination that Anthomyiidae can provide [42]. The other dipteran families were represented at lower percentages (<15%); among them, Sphaeroceridae were the most abundant, and all specimens but one belonged to two species: *Leptocera caenosa* (Rondani, 1880) and *Spelobia* cf. *clunipes* (Meigen, 1830). The former is mainly considered a synanthropic species, and its presence could be due to mountain huts at the sampling sites; however, both species were observed in burrows of mammals [79]. Here, too, however, their presence on flowers could be due to basking or trophic activity, even though the latter seems to be less likely as they prefer to feed on decaying organic matter of animal or plant origin [80]. The second most abundant order was Hymenoptera, with species belonging to two groups: ants and parasitoid wasps. Among them, the most represented family was Formicidae, and in particular *Formica lemani* Bondroit, 1917, an active predacious, aphidicolous, and nectarivorous species, whose workers forage on the ground, flowers, and plants [43,81,82]. In the Alps, this species is common in the grasslands above the tree line, where carbohydrates are a limiting resource, and therefore, ants can visit flowers to look for these important nutrients [83,84]. We also detected many families of parasitoid wasps, whose adults can visit flowers to feed on nectar [85,86]. Moreover, considering that all specimens identified at sex level were females, it is possible that they were searching for a host. In particular, the most abundant family was Braconidae, with almost all species belonging to the subfamily Aphidiinae, which are exclusive parasitoids of aphids [87]. Among Hemiptera, we found only winged forms of aphids, most of which belonged to the genus *Cinara* Curtis, 1835. Owing to the very early moment of the season, it is likely that aphids reached these mountain sites as aeroplankton, as demonstrated in other Alpine environments [88]. In our case, aphids could represent an important feeding resource for predators such as *F. lemani* [84]. Regarding Thysanoptera, all samples belonged to Thripidae, which may use flowers as shelter and mating sites, as well as food resources [89,90,91]. Although their presence and abundance seem to indicate a relevant role of this taxon in the context of this Alpine environment, it is difficult to draw any conclusions about the biotic interactions in which they might be involved. Indeed, very few studies have investigated their role in natural ecosystems, but they are well known as economically significant crop pests, and, in this context, it was suggested that their presence on plants can impact the behaviour and performance of other visiting insects [92]. The presence of Coleoptera on *A. brevis* flowers was very limited and therefore probably accidental in most cases. Therefore, the arthropod community associated with *A. brevis* flowers seems quite complex, despite the early flowering season, with potentially interesting biotic interactions with the plant as well as among insects.

Considering the general abundance pattern, our research highlights the dominance of flies among the high-altitude flower visitors, as already found in other studies on the Alps [36,37,41,42,93,94] and hypothesised as a global feature [95]. However, three peculiar aspects emerge from our work. Firstly, among flies, we found an evident predominance of Anthomyiidae. This pattern was already observed at higher altitudes [36,37,93,96] but not at the same elevation at which we operated, where Muscidae and Empididae seem to predominate [36]. However, we did not detect these two latter families, except for a single specimen of Muscidae. This result highlights the peculiarity of the early season flower-visiting community. Secondly, bees (Hymenoptera: Apoidea: Anthophila) were not collected during timed observations, but only a few specimens were found during the free observations. Their presence is, therefore, rare in this period, while later in the season, they are more common at these altitudes, though not as abundant as flies [36]. Even for bees, our early season pattern seems to recall what happens at higher altitudes, where the presence of these insects is very limited [36]. Indeed, a global switch from bees to flies along elevation gradients suggests that temperature may be a limiting factor for bees in high-altitude habitats, a pattern that, in the future, could be impacted by climate change, with unknown effects on fly communities already present at higher elevations [95]. Finally, we identified a considerable share of parasitoid wasps. This finding did not emerge from previous studies in the Alpine environment. Indeed, most of the studies did not look for this taxon, and even when parasitoid wasps were considered, they were not detected in large numbers [37], with the partial exception of Ichneumonidae [42,93].

These findings underline the importance of focussing future research efforts also on neglected taxa such as flies and parasitoid wasps and not only on well-investigated taxa such as Hymenoptera Apoidea Anthophila, Diptera Syrphidae, and Lepidoptera, which certainly play a fundamental role in mountain ecosystems, but which in some contexts—such as ours—seem to be poorly represented. Although bees, even when numerically limited, can play an important role as pollinators due to their efficiency in pollen dispersal, other taxa might also represent keystones of biotic interactions and be strongly threatened by climate change. For instance, despite the importance of flies as pollinators, especially in mountain environments [95], these insects have received little consideration in the literature [77,97]. Moreover, little is known about parasitoid wasp–flower interactions and their interdependence, as only very few observations of parasitoid–flower interactions have been reported [86], although they might have crucial roles in ecosystem functioning that could be affected by climate change [98].

In addition to describing the flower-visiting arthropod community of an early flowering high-altitude Alpine plant, our study provides new insights into how micrometeorological conditions (i.e., temperature and wind speed) and other variables (i.e., time and number of flowers per plant) affect the presence, diversity, and abundance of flower-visiting arthropods. As expected in a cold environment during the early season, where temperature constitutes a limiting factor for flower-visitor activity, this variable had a significant, positive effect on the presence, diversity, and abundance of flower-visiting arthropods. However, although in the present context higher temperatures can increase the activity of the flower-visiting arthropods, climate warming could impose new physiological constraints in the future [99], limiting their performance at elevated temperatures currently not occurring in the mountain environment during the early season. We did not observe any significant effect of wind speed on the presence, diversity, and abundance of flower-visiting arthropods. To the best of our knowledge, our study is the first in which the effect of wind on flower visitors in the Alps was investigated, although the effect of wind speed on the activity and flight of insects has been evaluated in some previous papers, with mixed results [57,100,101,102,103,104,105,106]. Even though each taxon and species might respond differently to microclimatic conditions, it is possible to hypothesise that arthropods capable of living in an extreme environment might be somehow adapted to the wind. However, during our fieldwork, the wind speed was always lower than 4.4 m/s, and no extreme wind conditions occurred. Therefore, we cannot rule out that higher wind speed could affect the flower visitors’ activity since a nonlinear response of arthropods to wind speed cannot be excluded [100]. Moreover, as the wind speed can change rapidly, with gusts and sudden variations, especially in extreme environments such as mountain ridges, it might be interesting to more accurately evaluate its effect on flower visitors’ behaviour through video observations [45]. The number of flowers per plant did not affect any of the dependent variables considered; this might seem quite surprising, as other studies highlighted its positive effect on flower visits [107,108,109]. However, owing to the number of flowers in the plants studied (always ≥ 20), we could not detect whether plants with fewer flowers would receive fewer visits; indeed, the positive effect of the number of flowers on the number of visits per plant might be more pronounced for low flower numbers and less marked for plants with many flowers [110]. Regarding the daily pattern of flower-visiting arthropod activity, we observed a significant effect of quadratic time on both flower visitors’ presence and abundance but not on their diversity. This bimodal pattern, in our case with a reduction in presence and abundance in the central hours of the day, has also already been observed in some other studies in different environments for many brachyceran flies (the group to which most of the *A. brevis* dipteran flower visitors belonged), and also for some bees, ants, and beetles [77,111,112,113,114]. However, climate conditions can influence this pattern [77], which might, therefore, potentially be affected by climate change. The lack of effect of quadratic time on diversity might suggest that the time influences the activity of arthropods, but it does not have a differential effect on the detected flower-visiting taxa. These considerations, although preliminary, could represent a starting point for assessing the variables that can impact the activity of Alpine early season flower-visiting communities.

In conclusion, this study contributes to our understanding of the composition and response to environmental variables of high-altitude Alpine flower-visiting arthropod communities in the early season. These communities can impact ecosystem function and stability not simply due to pollination but also because arthropods are part of complex biotic interactions that, if modified, can lead to secondary extinctions [115], with effects also later in the season [116]. This research also provides baseline data, collected using replicable and standardised methods, that can be useful for further studies on the effects of climate change at high altitudes, the impact of which on arthropods can be considerable but is still largely unclear and unknown [11,60,117].

## Figures and Tables

**Table 1:** Flower-visiting arthropod sampling by timed observations. Year, site, day, time windows, number (N) of sampling sessions, hours (h) of timed observations per year, and number (N) of flowers at anthesis present per plant (letters indicate the identity of the plant) are reported.

Year	Site	Day	Time Windows	N of Sampling Sessions	h of Timed Observations per Year	N of Flowers at Anthesis (per Plant)
**2016**	SJP	25 May 2016	11.30–12.30	8	16	69 (A); 109 (B)
			13.30–14.30			
			15.30–16.30			
			17.30–18.30			
		26 May 2016	11.30–12.30	8		
			13.30–14.30			
			15.30–16.30			
			17.30–18.30			
**2017**	BEN	31 May 2017	11.00–12.00	8	16	37 (C); 25 (E)
			12.30–13.30			
			14.00–15.00			
			15.30–16.30			
		1 June 2017	11.00–12.00	8		
			12.30–13.30			
			14.00–15.00			
			15.30–16.30			
**2018**	BEN	9 June 2018	13.00–14.00	8	16	33 (F); 36 (G)
			14.30–15.30			
			16.00–17.00			
			17.30–18.30			
		10 June 2018	10.15–11.15	8		
			11.45–12.45			
			13.15–14.15			
			14.45–15.45			
**2019**	SJP	4 June 2019	10.30–11.30	6	6	20 (H); 25 (I)
			12.00–13.00			
			15.00–16.00			

**Table 2:** Arthropods sampled by timed observations: number (N) of sampled specimens, captures per hour (mean ± SEM) and number of orders and families, reported for each year and site and overall.

	SJP 2016	BEN 2017	BEN 2018	SJP 2019	Total
**N of sampled specimens**	58	14	35	33	140
**Captures per hour (mean ± SEM)**	3.6 ± 0.5	0.9 ± 0.3	2.2 ± 0.6	5.5 ± 1.3	2.6 ± 0.3
**N of orders**	5	6	5	7	9
**N of families**	17	9	9	17	33

## Data Availability

The data presented in this study are available on request from the corresponding author.

## Supplementary Materials

The following supporting information can be downloaded at: https://www.mdpi.com/article/10.3390/insects13040393/s1, Figure S1: Boxplots of the micrometeorological conditions recorded during the timed observations, Table S1: Taxonomists who identified the sampled arthropods and their affiliation, Table S2: Molecular identification through COI barcoding, Table S3: Flower-visiting arthropods sampled during the timed observations, Table S4: Flower-visiting arthropods sampled during the free observations, Table S5: Model performances, Table S6: Effect of micrometeorological and context variables on flying flower visitors. References [118,119,120] are cited in the supplementary materials.
