# Supplementary material for: The Early Season Community of Flower-Visiting Arthropods in a High-Altitude Alpine Environment
Source: Insects. 2022 Apr 16;13(4):393. doi: 10.3390/insects13040393 (PMC9032982; doi:10.3390/insects13040393)
Supplement: Supplementary file 1 [file insects-13-00393-s001.zip › insects-1652305-supplementary.pdf]

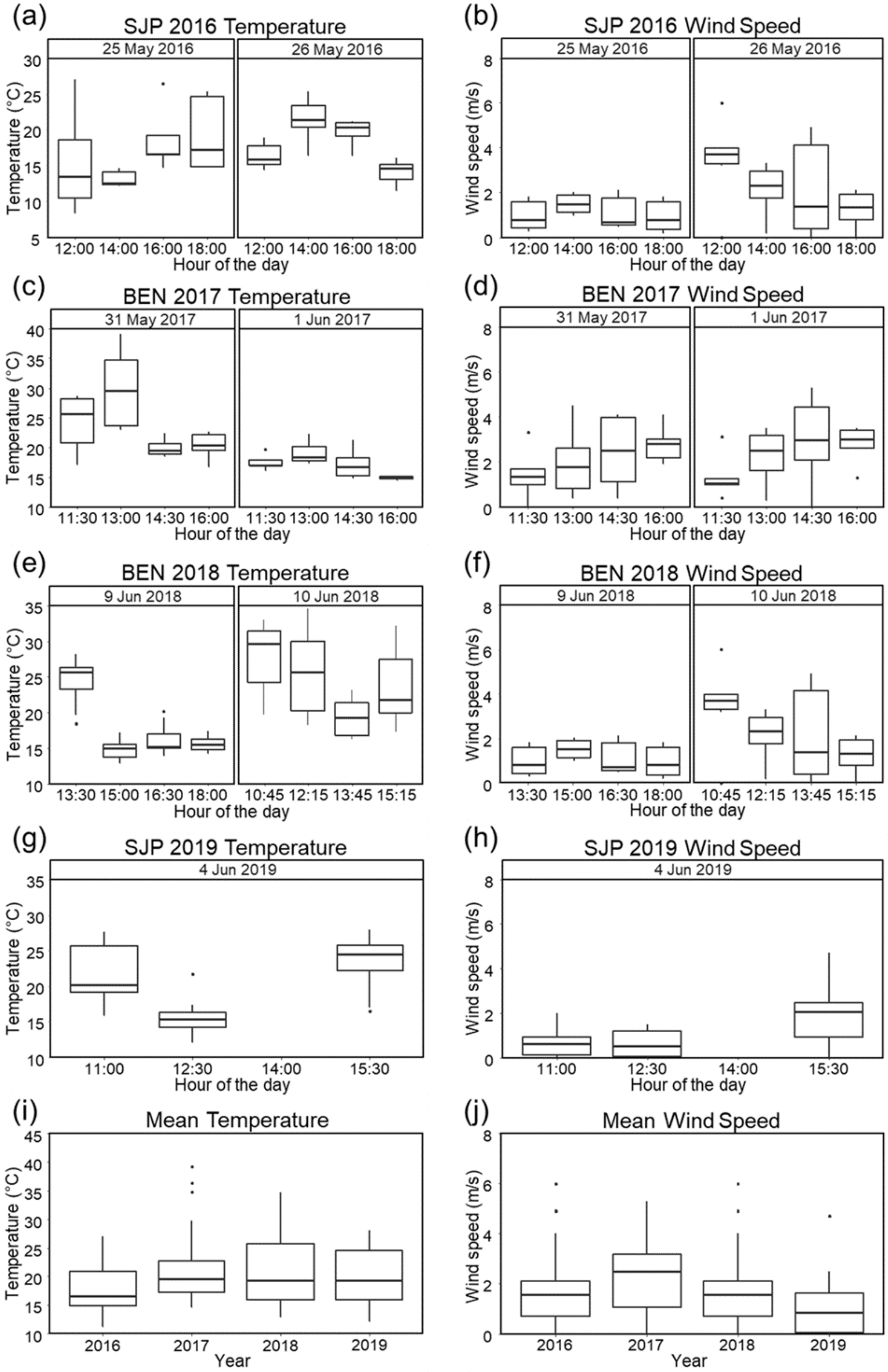

Figure S1. Boxplots of the micrometeorological conditions recorded during the timed observations: temperature and wind speed at SJP 2016 (a, b), BEN 2017 (c, d), BEN 2018 (e, f) and SJP 2019 (g, h) and mean temperature (i) and wind speed (j) through all years and sites.

Table S1. Taxonomists who identified the sampled arthropods and their affiliation.

Taxon	Taxonomist (affiliation)
**ARACHNIDA**	
**Araneae**	Paolo Pantini (Zoology of Invertebrate Section, Civic Museum of Natural Science Enrico Caffi, Bergamo, Italy)
**Oribatida**	Massimo Plumari (Museo Civico di Lentate sul Seveso, Lentate sul Seveso, Italy)
**HEXAPODA**	
**Coleoptera**	Mauro Gobbi
**Coleoptera, Chrysomelidae**	Matteo Montagna (Department of Agricultural and Environmental Sciences - Production Landscape Agroenergy, University of Milan, Milan, Italy)
**Coleoptera, Meloidae**	Marco Alberto Bologna (Department of Science, Roma Tre University, Rome, Italy)
**Collembola**	Pietro Paolo Fanciulli (Department of Life Sciences, University of Siena, Siena, Italy)<br>Barbara Valle (Department of Biosciences, University of Milan, Milan, Italy)
**Diptera**	Daniele Avesani
**Diptera, Agromyzidae**	Miloš Černý (Independent Taxonomist, Halenkovice, Czech Republic)
**Diptera, Anthomyiidae**	Verner Michelsen
**Diptera, Chironomidae**	Valeria Lencioni (Research and Museum Collections Office, Climate and Ecology Unit, MUSE - Science Museum, Trento, Italy)
**Diptera, Chloropidae**	Emilia Petrovna Nartshuk (Zoological Institute, Russian Academy of Sciences, Saint Petersburg, Russia)
**Diptera, Drosophilidae**	Gerhard Bächli (Department of Evolutionary Biology and Environmental Studies, University of Zurich, Zurich, Switzerland)
**Diptera, Phoridae**	Ronald Henry Lambert Disney (Department of Zoology, University of Cambridge, Cambridge, United Kingdom)
**Diptera, Scathophagidae**	Andrey Ozerov (Zoological Museum, Moscow State University, Moscow, Russia)
**Diptera, Sphaeroceridae**	Jindřich Roháček (Department of Entomology, Silesian Museum, Opava, Czech Republic)
**Diptera, Syrphidae**	Daniele Sommaggio (Department of Agricultural and Food Sciences, University of Bologna, Bologna, Italy)
**Hemiptera, Auchenorrhyncha**	Davide Scaccini (Department of Agronomy Food Natural Resources Animals and Environment, University of Padua, Padua, Italy)
**Hemiptera, Sternorrhyncha (2016, 2017, 2018)**	Lidia Limonta (Department of Food Environmental and Nutritional Sciences, University of Milan, Italy)
**Hemiptera, Sternorrhyncha (2019)**	Alice Casiraghi (Department of Evolutionary Biology Ecology and Environmental Sciences, University of Barcelona, Spain; Institute for Integrative Systems Biology, University of Valencia, Spain)<br>Nicolas Pérez Hidalgo (Institute for Integrative Systems Biology, University of Valencia, Spain; Arthropod Department, Museum of Natural Sciences of Barcelona, Spain)
**Hymenoptera (2016)**	Davide Dal Pos (Department of Biology, University of Central Florida, Orlando, United States)
**Hymenoptera (2017, 2018, 2019)**	Fabrizio Rigato (Sezione di Entomologia, Museo Civico di Storia Naturale, Milan, Italy)
**Hymenoptera, Apoidea (2016)**	Marco Bonelli<br>Mauro Gobbi
**Hymenoptera, Apoidea (2017, 2019)**	Andree Cappellari (Department of Agronomy, Food, Natural Resources, Animals and Environment, University of Padua, Padua, Italy)
**Hymenoptera, Braconidae**	Vladimir Žikić (Department of Biology and Ecology, University of Niš, Niš, Serbia)<br>Željko Tomanović (Department of Invertebrate Zoology and Entomology, University of Belgrade, Belgrade, Serbia)
**Hymenoptera, Eulophidae**	Alex Gumovsky (Department of Taxonomy of Entomophagous Insects and Ecological Principles of Biocontrol, National Academy of Sciences of Ukraine, Kyiv, Ukraine)
**Hymenoptera, Formicidae**	Fabrizio Rigato (Entomology Section, Milan Natural History Museum, Milan, Italy)
**Hymenoptera, Ichneumonidae**	Filippo Di Giovanni (Department of Agriculture Food and Environment, University of Pisa, Pisa, Italy)
**Hymenoptera, Mymaridae**	Riccardo Jesu (Department of Agriculture, University of Naples Federico II, Italy)
**Hymenoptera, Torymidae**	Adriano Cazzuoli (Independent Taxonomist, Modena, Italy)
**Lepidoptera**	Marco Bonelli
**Psocoptera**	Rinaldo Nicoli Aldini (Department of Sustainable Crop Production, Università Cattolica del Sacro Cuore, Piacenza-Cremona, Italy)
**Thysanoptera**	Barbara Conti (Department of Agriculture Food and Environment, University of Pisa, Pisa, Italy)

Table S2. Molecular identification through COI barcoding. Taxa and sexes, specimen ID and GenBank accession number, primer sets (names, sequences, lengths) and their references, PCR conditions, and the size of the amplicons obtained are reported.

**Taxon (sex)**	**Specimen ID (GenBank accession number)**	**Primer set (names, sequences, lengths)**	**Reference**	**PCR protocol**	**Amplicon size**
Hymenoptera Apidae *Bombus* sp. (male)	2017V02 (OM220090)	Bombus F GGATCNCCWGATATAGCWTTTCC (23)<br>Bombus R TGCAGANGTAAAATAAGCTCGTG (23)	designed with Primer-BLAST [118] and adapted	94°C for 3’, followed by 35 cycles at 94°C for 30’’, 51°C for 30’’, and 72°C for 45’’, and then 10’ at 72°C	387 bp
Thysanoptera Thripidae (unknown)	G02* (OM220091)	LCO1490 GGTCAACAAATCATAAAGATATTGG (25)<br>HCO2198 TAAACTTCAGGGTGACCAAAAAATCA (25)	[119]	94°C for 3’, followed by 35 cycles at 94°C for 30’’, 53°C for 30’’, and 72°C for 45’’, and then 10’ at 72°C	649 bp
Diptera Sciaridae (females)	H04 (OM220092)<br>H06 (OM220093)<br>2018VV18 (OM220094)<br>2018VV19 (OM220095)<br>2018VV27 (OM220096)				

* Damaged specimen.

Table S3. Flower-visiting arthropods sampled during the timed observations. Site and year, specimen ID, order, family, genus, species, stage (A=adult, J=juvenile) and repository are reported. Blank lines mean that taxonomic identification was not achieved. Nomenclature is according to Fauna Europaea [120].

Site and Year	ID	Order	Family	Genus	Species	Author	Stage	Repository*
SJP2016	A01	Psocoptera	Ectopsocidae	*Ectopsocus*		McLachlan, 1899	A	CSCU
SJP2016	A02	Diptera	Drosophilidae				A	MUSE
SJP2016	A03	Diptera	Sphaeroceridae	*Leptocera*	*Leptocera caenosa*	(Rondani, 1880)	A	SM
SJP2016	A04	Diptera	Sphaeroceridae	*Leptocera*	*Leptocera caenosa*	(Rondani, 1880)	A	SM
SJP2016	A05	Diptera	Sciaridae				A	MUSE
SJP2016	A06	Diptera	Anthomyzidae				A	MUSE
SJP2016	A07	Diptera	Sphaeroceridae	*Spelobia*	*Spelobia* cf. *clunipes*	(Meigen, 1830)	A	MUSE
SJP2016	A08	Diptera	Chironomidae				A	MUSE
SJP2016	A10	Diptera	Phoridae	*Megaselia*	*Megaselia rufipes*	(Meigen, 1804)	A	MUSE
SJP2016	A11	Hemiptera	Aphididae	*Chaitophorus*		Koch, 1854	A	UNIMI A
SJP2016	AA01	Hymenoptera	Eulophidae	*Baryscapus*		Forster, 1856	A	UNIMI B
SJP2016	AA02	Hymenoptera	Braconidae	*Aphidius*		Nees, 1818	A	UN
SJP2016	AA03	Hymenoptera	Braconidae	*Aphidius*	*Aphidius ervi*	Haliday, 1834	A	UNIMI B
SJP2016	AA04	Hymenoptera	Eulophidae	*Baryscapus*		Forster, 1856	A	UNIMI B
SJP2016	AA05	Hymenoptera	Braconidae	*Aphidius*	*Aphidius ervi*	Haliday, 1834	A	UN
SJP2016	AA06	Hemiptera	Aphididae	*Cinara*		Curtis, 1835	A	UNIMI A
SJP2016	AA07	Hymenoptera	Formicidae	*Formica*	*Formica lugubris*	Zetterstedt, 1838	A^{wg}	UNIMI B
SJP2016	AA08	Diptera	Sepsidae				A	MUSE
SJP2016	AA09	Diptera	Sphaeroceridae	*Spelobia*	*Spelobia* cf. *clunipes*	(Meigen, 1830)	A	MUSE
SJP2016	AA10	Hymenoptera	Braconidae	*Aphidius*	*Aphidius ervi*	Haliday, 1834	A	UN
SJP2016	AA11	Diptera	Sphaeroceridae	*Leptocera*	*Leptocera caenosa*	(Rondani, 1880)	A	SM
SJP2016	AA12	Hymenoptera	Ichneumonidae	*Aclastus*		Forster, 1869	A	UNIPI
SJP2016	AA13	Hymenoptera	Eulophidae	*Baryscapus*		Forster, 1856	A	UNIMI B
SJP2016	AA14	Diptera	Sciaridae				A	MUSE
SJP2016	AA15	Diptera	Sciaridae				A	MUSE
SJP2016	B01a	Hymenoptera	Formicidae	*Formica*	*Formica* (*Serviformica*) *lemani*	Bondroit, 1917	A	UNIMI B
SJP2016	B01b	Hymenoptera	Formicidae	*Formica*	*Formica* (*Serviformica*) *lemani*	Bondroit, 1917	A	UNIMI B
SJP2016	B01c	Hymenoptera	Formicidae	*Formica*	*Formica* (*Serviformica*) *lemani*	Bondroit, 1917	A	UNIMI B
SJP2016	B02	Hemiptera	Aphididae	*Cinara*		Curtis, 1835	A	UNIMI A
SJP2016	B03	Diptera	Lonchopteridae				A	MUSE
SJP2016	B04	Hymenoptera	Formicidae	*Formica*	*Formica* (*Serviformica*) *lemani*	Bondroit, 1917	A	UNIMI B
SJP2016	B05	Hymenoptera	Formicidae	*Formica*	*Formica* (*Serviformica*) *lemani*	Bondroit, 1917	A	UNIMI B
SJP2016	B06	Diptera	Chironomidae				A	MUSE
SJP2016	B07	Hemiptera	Aphididae	*Chaitophorus*		Koch, 1854	A	UNIMI A
SJP2016	B08	Diptera	Sphaeroceridae	*Leptocera*	*Leptocera caenosa*	(Rondani, 1880)	A	SM
SJP2016	B09	Hemiptera	Aphididae	*Cinara*		Curtis, 1835	A	UNIMI A
SJP2016	B10	Hemiptera	Aphididae	*Cinara*		Curtis, 1835	A	UNIMI A
SJP2016	B11	Hymenoptera	Formicidae	*Formica*	*Formica* (*Serviformica*) *lemani*	Bondroit, 1917	A	UNIMI B
SJP2016	B12	Hymenoptera	Formicidae	*Formica*	*Formica* (*Serviformica*) *lemani*	Bondroit, 1917	A	UNIMI B
SJP2016	B13	Hymenoptera	Braconidae	*Chorebus*		Haliday, 1833	A	UN

SJP2016	B14	Hymenoptera	Formicidae	*Formica*	*Formica* (*Serviformica*) *lemani*	Bondroit, 1917	A	UNIMI B
SJP2016	BB01	Hymenoptera	Braconidae	*Aphidius*	*Aphidius ervi*	Haliday, 1834	A	UN
SJP2016	BB02	Diptera	Chloropidae				A	MUSE
SJP2016	BB03	Hymenoptera	Formicidae	*Formica*	*Formica* (*Serviformica*) *lemani*	Bondroit, 1917	A	UNIMI B
SJP2016	BB04	Diptera	Sphaeroceridae	*Leptocera*	*Leptocera caenosa*	(Rondani, 1880)	A	SM
SJP2016	BB05	Hymenoptera	Braconidae	*Diaeretiella*		Stary, 1960	A	UN
SJP2016	BB06	Hymenoptera	Formicidae	*Formica*	*Formica* (*Serviformica*) *lemani*	Bondroit, 1917	A	UNIMI B
SJP2016	BB07	Hemiptera	Aphididae	*Cinara*		Curtis, 1835	A	UNIMI A
SJP2016	BB08	Hymenoptera	Braconidae	*Aphidius*		Nees, 1818	A	UN
SJP2016	BB09	Hymenoptera	Torymidae	*Torymus*	*Torymus baudysi*	Boucek, 1954	A	AC – pc
SJP2016	BB10	Diptera	Sciaridae				A	MUSE
SJP2016	BB11	Hemiptera	Aphididae	*Chaitophorus*		Koch, 1854	A	UNIMI A
SJP2016	BB12	Diptera	Chloropidae				A	MUSE
SJP2016	BB13	Hymenoptera	Braconidae	*Aphidius*	*Aphidius ervi*	Haliday, 1834	A	UN
SJP2016	BB14	Diptera	Sciaridae				A	MUSE
SJP2016	BB15	Hymenoptera	Braconidae	*Aphidius*	*Aphidius ervi*	Haliday, 1834	A	UN
SJP2016	BB15bis	Coleoptera	Staphylinidae	*Amphichroum*	*Amphichroum canaliculatum*	(Erichson, 1840)	A	MUSE
SJP2016	BB16	Diptera	Sphaeroceridae	*Opalimosina*	*Opalimosina* (*Opalimosina*) *mirabilis*	(Collin, 1902)	A	SM
BEN2017	C01	Thysanoptera	Thripidae	*Thrips*	*Thrips vulgatissimus*	Haliday, 1836	A	UNIPI
BEN2017	C02	Diptera	Chironomidae	*Smittia*		Holmgren, 1869	A	MUSE
BEN2017	C03	Hymenoptera	Mymaridae	*Gonatocerus*		Nees, 1834	A	MSN
BEN2017	C04	Diptera	Agromyzidae	*Chromatomyia*	*Chromatomyia fuscula*	(Zetterstedt, 1838)	A	MC – pc
BEN2017	C05	Coleoptera	Meloidae	*Meloe*	*Meloe* (*Meloe*) *violaceus*	Marsham, 1802	J	UNIMI B
BEN2017	C06	Oribatida					A	UNIMI B
BEN2017	C07	Thysanoptera	Thripidae	*Thrips*	*Thrips vulgatissimus*	Haliday, 1836	A	UNIPI
BEN2017	C08	Thysanoptera	Thripidae	*Thrips*	*Thrips vulgatissimus*	Haliday, 1836	A	UNIPI
BEN2017	E01	Hymenoptera	Formicidae	*Formica*	*Formica* (*Serviformica*) *lemani*	Bondroit, 1917	A	UNIMI B
BEN2017	E02	Hemiptera	Aphididae				A	UNIMI A
BEN2017	E03	Hemiptera	Aphididae	*Cinara*		Curtis, 1835	A	UNIMI A
BEN2017	CC01	Hymenoptera	Formicidae	*Lasius*	*Lasius* (*Dendrolasius*) *fuliginosus*	(Latreille, 1798)	A^{wg}	UNIMI B
BEN2017	CC02	Thysanoptera	Thripidae	*Thrips*	*Thrips vulgatissimus*	Haliday, 1836	A	UNIPI
BEN2017	EE01	Hymenoptera	Braconidae	*Aphidius*	*Aphidius avenae*	Haliday, 1834	A	UNIMI B
BEN2018	F01	Diptera	Anthomyiidae	*Delia*	*Delia platura*	(Meigen, 1826)	A	MUSE
BEN2018	F02	Diptera	Anthomyiidae	*Delia*	*Delia platura*	(Meigen, 1826)	A	MUSE
BEN2018	F03	Diptera	Anthomyiidae	*Delia*	*Delia platura*	(Meigen, 1826)	A	MUSE
BEN2018	G01	Araneae	Linyphiidae	*Agyneta*		Hull, 1911	A	UNIMI B
BEN2018	G02	Thysanoptera	Thripidae	*Thrips*	*Thrips vulgatissimus*	Haliday, 1836	A	BARCODING
BEN2018	G03	Diptera	Anthomyiidae	*Delia*	*Delia platura*	(Meigen, 1826)	A	MUSE
BEN2018	G04	Diptera	Anthomyiidae	*Delia*	*Delia platura*	(Meigen, 1826)	A	MUSE
BEN2018	G05	Diptera	Anthomyiidae	*Delia*	*Delia platura*	(Meigen, 1826)	A	MUSE
BEN2018	FF05	Diptera	Anthomyiidae	*Delia*	*Delia platura*	(Meigen, 1826)	A	MUSE
BEN2018	FF06	Diptera	Anthomyiidae	*Delia*	*Delia platura*	(Meigen, 1826)	A	MUSE
BEN2018	FF07	Diptera	Chloropidae	*Oscinella*	*Oscinella* (*Oscinella*) *frit*	(Linnaeus. 1758)	A	UNIMI B

BEN2018	FF09	Coleoptera	Coccinellidae	*Coccinella*	*Coccinella septempunctata*	Linnaeus, 1758	A	MUSE
BEN2018	FF10	Hymenoptera	Formicidae	*Formica*	*Formica* (*Serviformica*) *lemani*	Bondroit, 1917	A	MSN
BEN2018	FF11	Diptera	Anthomyiidae	*Delia*	*Delia platura*	(Meigen, 1826)	A	MUSE
BEN2018	GG01	Diptera	Anthomyiidae	*Delia*	*Delia platura*	(Meigen, 1826)	A	MUSE
BEN2018	GG02	Diptera	Anthomyiidae	*Delia*	*Delia platura*	(Meigen, 1826)	A	MUSE
BEN2018	GG03	Diptera	Anthomyiidae	*Delia*	*Delia platura*	(Meigen, 1826)	A	UNIMI B
BEN2018	GG04	Hymenoptera	Mymaridae				A	MSN
BEN2018	GG05	Diptera	Anthomyiidae	*Delia*	*Delia platura*	(Meigen, 1826)	A	MUSE
BEN2018	GG06	Diptera	Anthomyiidae	*Delia*	*Delia platura*	(Meigen, 1826)	A	MUSE
BEN2018	GG07	Diptera	Anthomyiidae	*Paregle*	*Paregle coerulescens*	(Strobl, 1893)	A	MUSE
BEN2018	GG08	Diptera	Anthomyiidae	*Paregle*	*Paregle coerulescens*	(Strobl, 1893)	A	MUSE
BEN2018	GG09	Diptera	Anthomyiidae	*Delia*	*Delia platura*	(Meigen, 1826)	A	MUSE
BEN2018	GG10	Diptera	Chironomidae	*Camptocladius*	*Camptocladius* cf. *stercorarius*	(Da Geer, 1776)	A	MUSE
BEN2018	GG11	Diptera	Anthomyiidae	*Delia*	*Delia platura*	(Meigen, 1826)	A	MUSE
BEN2018	GG12	Diptera	Anthomyiidae	*Paregle*	*Paregle coerulescens*	(Strobl, 1893)	A	MUSE
BEN2018	GG13	Diptera	Anthomyiidae	*Pegoplata*	*Pegoplata aestiva*	Meigen, 1826	A	MUSE
BEN2018	GG14	Diptera	Drosophilidae	*Scaptomyza*	*Scaptomyza pallida*	(Zetterstedt, 1847)	A	UZ
BEN2018	GG15	Diptera	Anthomyiidae	*Paregle*	*Paregle coerulescens*	(Strobl, 1893)	A	MUSE
BEN2018	GG16	Diptera	Anthomyiidae	*Paregle*	*Paregle coerulescens*	(Strobl, 1893)	A	MUSE
BEN2018	GG17	Diptera	Anthomyiidae	*Paregle*	*Paregle coerulescens*	(Strobl, 1893)	A	MUSE
BEN2018	GG18	Diptera	Anthomyiidae	*Paregle*	*Paregle coerulescens*	(Strobl, 1893)	A	MUSE
BEN2018	GG19	Diptera	Anthomyiidae	*Paregle*	*Paregle coerulescens*	(Strobl, 1893)	A	MUSE
BEN2018	GG20	Diptera	Anthomyiidae	*Paregle*	*Paregle coerulescens*	(Strobl, 1893)	A	MUSE
BEN2018	GG21	Diptera	Anthomyiidae	*Paregle*	*Paregle coerulescens*	(Strobl, 1893)	A	MUSE
SJP2019	I01	Hymenoptera	Formicidae	*Formica*	*Formica* (*Serviformica*) *lemani*	Bondroit, 1917	A	UNIMI B
SJP2019	I02	Diptera	Cecidomyiidae				A	MS – pc
SJP2019	I03	Hymenoptera	Formicidae	*Formica*	*Formica* (*Serviformica*) *lemani*	Bondroit, 1917	A	UNIMI B
SJP2019	I04	Hymenoptera	Formicidae	*Formica*	*Formica* (*Serviformica*) *lemani*	Bondroit, 1917	A	UNIMI B
SJP2019	I05	Hymenoptera	Formicidae	*Formica*	*Formica* (*Serviformica*) *lemani*	Bondroit, 1917	A	UNIMI B
SJP2019	I06	Hymenoptera	Formicidae	*Leptothorax*	*Leptothorax acervorum*	(Fabricius, 1793)	A	UNIMI B
SJP2019	I07	Hymenoptera	Formicidae	*Formica*	*Formica* (*Serviformica*) *lemani*	Bondroit, 1917	A	UNIMI B
SJP2019	I08	Hymenoptera	Formicidae	*Formica*	*Formica* (*Serviformica*) *lemani*	Bondroit, 1917	A	UNIMI B
SJP2019	I09	Diptera	Chironomidae	*Parorthocladius*	*Parorthocladius* cf. *nudipennis*	(Kieffer, 1908)	A	MUSE
SJP2019	I10	Hymenoptera	Braconidae				A	MSN
SJP2019	I11	Coleoptera	Chrysomelidae	*Plagiosterna*	*Plagiosterna aenea*	(Linnaeus, 1758)	A	UNIMI B
SJP2019	I12	Thysanoptera	Thripidae	*Thrips*	*Thrips vulgatissimus*	Haliday, 1836	A	UNIPI
SJP2019	I13	Hymenoptera	Formicidae	*Formica*	*Formica* (*Serviformica*) *lemani*	Bondroit, 1917	A	UNIMI B
SJP2019	I14	Hymenoptera	Formicidae	*Formica*	*Formica* (*Serviformica*) *lemani*	Bondroit, 1917	A	UNIMI B
SJP2019	I15	Oribatida					A	UNIMI B
SJP2019	I16	Collembola	Entomobryidae	*Orchesella*	*Orchesella arcuata*	Lindenmann, 1950	A	UNIMI B
SJP2019	I17	Hymenoptera	Scelionidae				A	UNIMI B
SJP2019	I18	Hymenoptera	Formicidae	*Formica*	*Formica* (*Serviformica*) *lemani*	Linnaeus, 1758	A	UNIMI B
SJP2019	I19	Hymenoptera	Encyrtidae				A	UNIMI B

SJP2019	H01	Diptera	Phoridae	*Phora*		Latreille, 1796	A	UC
SJP2019	H02	Hymenoptera	Braconidae				A	MSN
SJP2019	H03	Hymenoptera	Megaspilidae				A	MSN
SJP2019	H04	Diptera	Sciaridae	*Austrosciara*	*Austrosciara hyalipennis*	(Meigen, 1804)	A	BARCODING
SJP2019	H05	Hemiptera	Aphididae	*Brachycaudus*		Van Der Goot, 1913	A	NPH – pc
SJP2019	H06	Diptera	Sciaridae	*Austrosciara*	*Austrosciara hyalipennis*	(Meigen, 1804)	A	BARCODING
SJP2019	H07	Diptera	Chironomidae	*Parorthocladius*	*Parorthocladius* cf. *nudipennis*	(Kieffer, 1908)	A	MUSE
SJP2019	H08	Hymenoptera	Braconidae				A	MSN
SJP2019	H09	Hymenoptera	Figitidae				A	MSN
SJP2019	H10	Thysanoptera	Thripidae	*Thrips*	*Thrips vulgatissimus*	Haliday, 1836	A	UNIPI
SJP2019	H11	Hymenoptera	Encyrtidae				A	MSN
SJP2019	H12	Hymenoptera	Pteromalidae				A	MSN
SJP2019	H13	Hymenoptera	Ceraphronidae				A	MSN
SJP2019	H14	Hymenoptera	Megaspilidae				A	MSN

* Repository acronyms: AC – pc: Adriano Cazzuoli – Personal Collection (Italy); CSCU: Department of Sustainable Crop Production, Catholic University of the Sacred Heart (Italy); MC – pc: Miloš Černý – Personal Collection (Czech Republic); MS – pc: Marcela Skuhravá – Personal collection (Czech Republic); MSN: Entomology Section, Milan Natural History Museum (Italy); MUSE: Research and Museum Collections Office Climate and Ecology Unit, MUSE – Science Museum (Italy); NPH – pc: Nicolás Pérez Hidalgo – Personal Collection (Spain); SM: Department of Entomology – Silesian Museum (Czech Republic); UC: Department of Zoology, University of Cambridge (United Kingdom); UN: Department of Biology and Ecology, University of Niš (Serbia); UNIMI A: Department of Food Environmental and Nutritional Sciences, University of Milan (Italy); UNIMI B: Department of Biosciences, University of Milan (Italy); UNIPI: Department of Agriculture Food and Environment, University of Pisa (Italy); UZ: Department of Evolutionary Biology and Environmental Studies, University of Zurich (Switzerland). ^{wf} = winged gyne

Table S4. Flower-visiting arthropods sampled during the free observations. Site and year, specimen ID, order, family, genus, species, stage (A=adult, J=juvenile) and repository are reported. Blank lines mean that taxonomic identification was not achieved. Nomenclature is according to Fauna Europaea [120].

Site and Year	ID	Order	Family	Genus	Species	Author	Stage	Repository
SPJ2016	2016V01	Hymenoptera	Ichneumonidae	*Probles*	*Probles* (*Microdiaparsis*) *caudiculatus*	Khalaim, 2007	A	UNIPI
SPJ2016	2016V02	Diptera	Phoridae	*Megaselia*	*Megaselia rufipes*		A	MUSE
SPJ2016	2016V03	Diptera	Ephydridae				A	MUSE
SPJ2016	2016V04	Hymenoptera	Braconidae	*Aphidius*	*Aphidius ervi*	Haliday, 1834	A	UN
SPJ2016	2016V05	Diptera	Anthomyiidae				A	MUSE
SPJ2016	2016V06	Hymenoptera	Torymidae	*Torymus*	*Torymus baudysi*	Boucek, 1954	A	AC – pc
SPJ2016	2016V07	Hemiptera	Aphididae	*Cinara*		Curtis, 1835	A	UNIMI A
SPJ2016	2016V08	Hymenoptera	Braconidae	*Aphidius*	*Aphidius ervi*	Haliday, 1834	A	UN
SPJ2016	2016V09	Hymenoptera	Apidae	*Bombus*	*Bombus lapidarius*	(Linnaeus, 1758)	A	MUSE
SPJ2016	2016V10	Diptera	Muscidae	*Helina*		Robineau-Desvoidy, 1830	A	MUSE
SPJ2016	2016V11	Hymenoptera	Torymidae	*Torymus*	*Torymus baudysi*	Boucek, 1954	A	AC – pc
SPJ2016	2016V12	Diptera	Sciaridae				A	MUSE
SPJ2016	2016VV01	Lepidoptera	Nymphalidae	*Aglais*	*Aglais urticae*	(Linnaeus, 1758)	A	UNIMI B
SPJ2016	2016VV02	Hymenoptera	Eulophidae	*Necremnus*	*Necremnus* cf. *cosconius*	(Walker, 1839)	A	UNIMI B
SPJ2016	2016VV03	Diptera	Anthomyiidae				A	MUSE
SPJ2016	2016VV04	Hymenoptera	Apidae	*Bombus*	*Bombus lapidarius*	(Linnaeus, 1758)	A	MUSE
SPJ2016	2016VV05	Diptera	Agromyzidae				A	MUSE
SPJ2016	2016VV06	Hymenoptera	Torymidae	*Torymus*	*Torymus baudysi*	Boucek, 1954	A	AC – pc
SPJ2016	2016VV07	Coleoptera	Curculionidae	*Orchestes*	*Orchestes* (*Salius*) *fagi*	(Linnaeus, 1758)	A	MUSE
SPJ2016	2016VV08	Hymenoptera	Torymidae	*Torymus*	*Torymus baudysi*	Boucek, 1954	A	AC – pc
SPJ2016	2016VV09	Hymenoptera	Torymidae	*Torymus*	*Torymus baudysi*	Boucek, 1954	A	AC – pc
SPJ2016	2016VV10	Coleoptera	Staphylinidae	*Amphichroum*	*Amphichroum canaliculatum*	(Erichson, 1840)	A	MUSE
SPJ2016	2016VV11	Coleoptera	Curculionidae	*Orchestes*	*Orchestes* (*Salius*) *fagi*	(Linnaeus, 1758)	A	MUSE
SPJ2016	2016VV12	Hemiptera	Aphididae	*Cinara*		Curtis, 1835	A	UNIMI A
BEN2017	2017V01	Coleoptera	Meloidae	*Meloe*	*Meloe* (*Meloe*) *violaceus*	Marsham, 1802	J	UNIMI B
BEN2017	2017V02	Hymenoptera	Apidae	*Bombus*	*Bombus pratorum*	(Linnaeus, 1761)	A	BARCODING
BEN2017	2017V03	Thysanoptera	Thripidae	*Thrips*	*Thrips vulgatissimus*	Haliday, 1836	A	UNIPI
BEN2017	2017V04	Hemiptera	Aphididae				A	UNIMI A
BEN2017	2017V05	Hymenoptera	Braconidae	*Aphidius*	*Aphidius avenae*	Haliday, 1834	A	UNIMI A
BEN2017	2017V06	Diptera	Anthomyiidae	*Delia*	*Delia platura*	(Meigen, 1826)	A	MUSE
BEN2017	2017V07	Diptera	Anthomyiidae	*Paregle*	*Paregle coerulescens*	(Strobl, 1893)	A	UNIMI B
BEN2017	2017V08	Hymenoptera	Halictidae	*Lasioglossum*		Curtis, 1833	A	UNIPD
BEN2017	2017V09	Hymenoptera	Halictidae	*Lasioglossum*		Curtis, 1833	A	UNIPD
BEN2017	2017V10	Diptera	Anthomyiidae	*Pegoplata*	*Pegoplata aestiva*	(Meigen, 1826)	A	MUSE
BEN2017	2017V10	Diptera	Anthomyiidae	*Pegoplata*	*Pegoplata aestiva*	Meigen, 1826	A	MUSE
BEN2017	2017V11	Diptera	Anthomyiidae	*Pegoplata*	*Pegoplata aestiva*	(Meigen, 1826)	A	MUSE
BEN2017	2017V12	Diptera	Anthomyiidae				A	MUSE
BEN2017	2017V13	Diptera	Anthomyiidae	*Pegoplata*	*Pegoplata aestiva*	(Meigen, 1826)	A	MUSE
BEN2017	2017V14	Diptera	Anthomyiidae	*Pegoplata*	*Pegoplata aestiva*	(Meigen, 1826)	A	MUSE
BEN2017	2017V15	Diptera	Anthomyiidae	*Zaphne*	*Zaphne frontata*	(Zetterstedt, 1838)	A	MUSE

BEN2017	2017VV01	Hymenoptera	Braconidae	*Aphidius*	*Aphidius avenae*	Haliday, 1834	A	UN
BEN2017	2017VV02	Hemiptera	Aphididae	*Eulachnus*		Del Guercio, 1909	A	UNIMI A
BEN2017	2017VV03	Thysanoptera	Thripidae	*Thrips*	*Thrips vulgatissimus*	Haliday, 1836	A	UNIPI
BEN2017	2017VV04	Thysanoptera	Thripidae	*Thrips*	*Thrips vulgatissimus*	Haliday, 1836	A	UNIPI
BEN2017	2017VV05	Thysanoptera	Thripidae	*Thrips*	*Thrips tabaci*	Lindeman, 1889	A	UNIPI
BEN2017	2017VV06	Diptera	Chloropidae	*Oscinella*	*Oscinella* (*Oscinella*) *frit*	(Linnaeus. 1758)	A	UNIMI B
BEN2017	2017VV07	Thysanoptera	Thripidae	*Thrips*	*Thrips vulgatissimus*	Haliday, 1836	A	UNIPI
BEN2017	2017VV08	Diptera	Anthomyiidae	*Delia*	*Delia platura*	(Meigen, 1826)	A	MUSE
BEN2017	2017VV09	Hymenoptera	Braconidae	*Aphidius*	*Aphidius ervi*	Haliday, 1834	A	UNIMI B
BEN2018	2018V01	Diptera	Anthomyiidae	*Zaphne*	*Zaphne frontata*	(Zetterstedt, 1838)	A	MUSE
BEN2018	2018V02	Hymenoptera	Figitidae				A	MSN
BEN2018	2018V03	Hymenoptera	Formicidae	*Myrmica*	*Myrmica sulcinodis*	Nylander, 1846	A	MSN
BEN2018	2018V04	Araneae	Linyphiidae	*Bathyphantes*	*Bathyphantes setiger*	F.O. Pickard-Cambridge, 1894	A	UNIMI B
BEN2018	2018V05	Diptera	Anthomyiidae	*Delia*	*Delia platura*	(Meigen, 1826)	A	MUSE
BEN2018	2018V06	Diptera	Anthomyiidae	*Paregle*	*Paregle coerulescens*	(Strobl, 1893)	A	MUSE
BEN2018	2018V07	Diptera	Anthomyiidae	*Paregle*	*Paregle coerulescens*	(Strobl, 1893)	A	MUSE
BEN2018	2018V08	Araneae	Linyphiidae	*Agyneta*			J	UNIMI B
BEN2018	2018V10	Diptera	Anthomyiidae	*Paregle*	*Paregle coerulescens*	(Strobl, 1893)	A	MUSE
BEN2018	2018VV01	Diptera	Anthomyiidae	*Delia*	*Delia platura*	(Meigen, 1826)	A	MUSE
BEN2018	2018VV02	Diptera	Anthomyiidae	*Delia*	*Delia platura*	(Meigen, 1826)	A	MUSE
BEN2018	2018VV03	Hymenoptera	Ichneumonidae				A	MSN
BEN2018	2018VV04	Diptera	Syrphidae	*Scaeva*	*Scaeva dignota*	(Rondani, 1857)	A	DS – pc
BEN2018	2018VV05	Hymenoptera	Formicidae	*Myrmica*	*Myrmica sulcinodis*	Nylander, 1846	A	MSN
BEN2018	2018VV06	Araneae	Linyphiidae	*Agyneta*			J	UNIMI B
BEN2018	2018VV07	Diptera	Anthomyiidae	*Delia*	*Delia platura*	(Meigen, 1826)	A	MUSE
BEN2018	2018VV08	Diptera	Scathophagidae	*Scathophaga*	*Scathophaga stercoraria*	(Linnaeus, 1758)	A	UNIMI B
BEN2018	2018VV09	Diptera	Anthomyiidae	*Delia*	*Delia platura*	(Meigen, 1826)	A	MUSE
BEN2018	2018VV10	Diptera	Anthomyiidae	*Delia*	*Delia platura*	(Meigen, 1826)	A	MUSE
BEN2018	2018VV11	Diptera	Anthomyiidae	*Delia*	*Delia platura*	(Meigen, 1826)	A	MUSE
BEN2018	2018VV12	Diptera	Anthomyiidae	*Delia*	*Delia platura*	(Meigen, 1826)	A	MUSE
BEN2018	2018VV13	Coleoptera	Staphylinidae	*Philonthus*	*Philonthus* (*Philonthus*) *frigidus*	Markel & Kiesenwetter, 1848	A	MUSE
BEN2018	2018VV14	Diptera	Anthomyiidae	*Paregle*	*Paregle coerulescens*	(Strobl, 1893)	A	MUSE
BEN2018	2018VV15	Hymenoptera	Ichneumonidae				A	MSN
BEN2018	2018VV16	Diptera	Anthomyiidae	*Delia*	*Delia platura*	(Meigen, 1826)	A	MUSE
BEN2018	2018VV17	Diptera	Anthomyiidae	*Delia*	*Delia platura*	(Meigen, 1826)	A	MUSE
BEN2018	2018VV18	Diptera	Sciaridae	*Corynoptera*	*Corynoptera trepida*	(Winnertz, 1867)	A	BARCODING
BEN2018	2018VV19	Diptera	Sciaridae	*Corynoptera*	*Corynoptera trepida*	(Winnertz, 1867)	A	BARCODING
BEN2018	2018VV20	Diptera	Anthomyiidae	*Zaphne*	*Zaphne frontata*	(Zetterstedt, 1838)	A	MUSE
BEN2018	2018VV21	Diptera	Anthomyiidae	*Delia*	*Delia platura*	(Meigen, 1826)	A	MUSE
BEN2018	2018VV22	Diptera	Anthomyiidae	*Paregle*	*Paregle coerulescens*	(Strobl, 1893)	A	MUSE
BEN2018	2018VV23	Diptera	Anthomyiidae	*Paregle*	*Paregle coerulescens*	(Strobl, 1893)	A	MUSE
BEN2018	2018VV24	Diptera	Anthomyiidae	*Paregle*	*Paregle coerulescens*	(Strobl, 1893)	A	MUSE
BEN2018	2018VV25	Collembola	Entomobryidae	*Orchesella*		Templeton, 1835	A	UNIMI B

BEN2018	2018VV26	Diptera	Anthomyiidae	*Delia*	*Delia platura*	(Meigen, 1826)	A	MUSE
BEN2018	2018VV27	Diptera	Sciaridae				A	BARCODING
BEN2018	2018VV28	Diptera	Anthomyiidae	*Paregle*	*Paregle coerulescens*	(Strobl, 1893)	A	MUSE
BEN2018	2018VV29	Diptera	Anthomyiidae	*Paregle*	*Paregle coerulescens*	(Strobl, 1893)	A	MUSE
BEN2018	2018VV30	Araneae	Theridiidae				J	UNIMI B
BEN2018	2018VV31	Diptera	Anthomyiidae	*Delia*	*Delia platura*	(Meigen, 1826)	A	MUSE
BEN2018	2018VV32	Diptera	Anthomyiidae	*Delia*	*Delia platura*	(Meigen, 1826)	A	MUSE
BEN2018	2018VV33	Diptera	Anthomyiidae	*Delia*	*Delia platura*	(Meigen, 1826)	A	MUSE
BEN2018	2018VIS01	Diptera	Anthomyiidae	*Delia*	*Delia platura*	(Meigen, 1826)	A	MUSE
BEN2018	2018VIS02	Diptera	Anthomyiidae	*Delia*	*Delia platura*	(Meigen, 1826)	A	MUSE
BEN2018	2018VIS03	Diptera	Anthomyiidae	*Delia*	*Delia platura*	(Meigen, 1826)	A	MUSE
BEN2018	2018VIS04	Diptera	Anthomyiidae	*Delia*	*Delia platura*	(Meigen, 1826)	A	MUSE
BEN2018	2018VIS05	Diptera	Anthomyiidae	*Delia*	*Delia platura*	(Meigen, 1826)	A	MUSE
BEN2018	2018VIS06	Diptera	Anthomyiidae	*Delia*	*Delia platura*	(Meigen, 1826)	A	MUSE
BEN2018	2018VIS07	Diptera	Anthomyiidae	*Delia*	*Delia platura*	(Meigen, 1826)	A	MUSE
BEN2018	2018VIS08	Diptera	Anthomyiidae	*Delia*	*Delia platura*	(Meigen, 1826)	A	MUSE
BEN2018	2018VIS09	Diptera	Anthomyiidae	*Delia*	*Delia platura*	(Meigen, 1826)	A	MUSE
BEN2018	2018VIS10	Diptera	Anthomyiidae	*Delia*	*Delia platura*	(Meigen, 1826)	A	MUSE
BEN2018	2018VIS11	Diptera	Anthomyiidae	*Delia*	*Delia platura*	(Meigen, 1826)	A	MUSE
BEN2018	2018VIS12	Diptera	Anthomyiidae	*Delia*	*Delia platura*	(Meigen, 1826)	A	MUSE
BEN2018	2018VIS13	Diptera	Anthomyiidae	*Delia*	*Delia platura*	(Meigen, 1826)	A	MUSE
BEN2018	2018VIS15	Diptera	Anthomyiidae	*Paregle*	*Paregle coerulescens*	(Strobl, 1893)	A	MUSE
BEN2018	2018VIS16	Diptera	Syrphidae	*Eristalis*	*Eristalis similis*	(Fallen, 1817)	A	DS – pc
BEN2018	2018VIS17	Hemiptera	Cicadellidae				J	UNIMI A
BEN2018	2018VIS18	Diptera	Anthomyiidae	*Delia*	*Delia platura*	(Meigen, 1826)	A	MUSE
BEN2018	2018VIS19	Diptera	Anthomyiidae	*Delia*	*Delia platura*	(Meigen, 1826)	A	MUSE
BEN2018	2018VIS20	Diptera	Anthomyiidae	*Paregle*	*Paregle coerulescens*	(Strobl, 1893)	A	MUSE
SJP2019	2019V01	Coleoptera	Cantharidae	*Cantharis*	*Cantharis* (*Cantharis*) *nigricans*	Müller, 1766	A	MUSE
SJP2019	2019V02	Hymenoptera	Tenthredinidae	*Dolerus*		Panzer, 1801	A	MSN
SJP2019	2019V03	Hymenoptera	Andrenidae	*Andrena*	*Andrena lapponica*	Zetterstedt, 1838	A	UNIPD
SJP2019	2019V04	Diptera	Anthomyiidae	*Delia*	*Delia platura*	(Meigen, 1826)	A	UNIMI B

* Repository acronyms: AC – pc: Adriano Cazzuoli – Personal Collection (Italy); DS – pc: Daniele Sommaggio – Personal Collection (Italy) MSN: Marcela Skuhravá – Personal collection (Czech Republic); MUSE: Research and Museum Collections Office Climate and Ecology Unit, MUSE – Science Museum (Italy); UN: Department of Biology and Ecology, University of Niš (Serbia); UNIMI A: Department of Food Environmental and Nutritional Sciences, University of Milan (Italy); UNIMI B: Department of Biosciences, University of Milan (Italy); UNIPD: Department of Agronomy Food Natural Resources Animals and Environment, University of Padova (Italy); UNIPI: Department of Agriculture Food and Environment, University of Pisa (Italy).

Table S5. Model performances. Likelihood ratio test and pseudo R^2.

Model	Likelihood ratio test			pseudo R^2	
	df	Explained deviance	p	pseudo R^2 value	Method
**Presence**	6	31.230	< 0.001	0.515	Tjur's R^2
**Diversity**	6	48.709	< 0.001	0.732	Nagelkerke's R^2
**Abundance**	6	60.923	< 0.001	0.711	Nagelkerke's R^2

Table S6. Effect of micrometeorological and context variables on flying flower visitors. Asterisk indicates statistically significant effects.

	**Presence**	**Diversity**	**Abundance**
**Temperature**	$\chi^2_1 = 9.66$, $p < 0.001$ *	$\chi^2_1 = 9.54$, $p < 0.01$ *	$\chi^2_1 = 19.82$, $p < 0.001$ *
**Wind Speed**	$\chi^2_1 = 0.22$, $p = 0.635$	$\chi^2_1 = 0.00$, $p = 0.965$	$\chi^2_1 = 0.89$, $p = 0.345$
**Time (quadratic)**	$\chi^2_1 = 2.35$, $p = 0.125$	$\chi^2_1 = 2.56$, $p = 0.109$	$\chi^2_1 = 5.20$, $p < 0.05$ *
**Number of flowers**	$\chi^2_1= 0.02$, $p = 0.885$	$\chi^2_1 = 0.09$, $p= 0.761$	$\chi^2_1 = 0.07$, $p = 0.789$
**Site**	$\chi^2_1 = 5.55$, $p < 0.05$ *	$\chi^2_1 = 25.24$, $p < 0.001$ *	$\chi^2_1 = 17.03$, $p < 0.001$ *
